# Protective versus Pathogenic Type I Interferon Responses during Virus Infections

**DOI:** 10.3390/v15091916

**Published:** 2023-09-13

**Authors:** Kwang Il Jung, Savannah McKenna, Vijayamahantesh Vijayamahantesh, Ying He, Bumsuk Hahm

**Affiliations:** Departments of Surgery & Molecular Microbiology and Immunology, University of Missouri, Columbia, MO 65212, USA; kjyzn@health.missouri.edu (K.I.J.); slmccr@missouri.edu (S.M.); vvpqn@health.missouri.edu (V.V.); hey1@health.missouri.edu (Y.H.)

**Keywords:** interferon, host defense, immune stimulation, immune suppression

## Abstract

Following virus infections, type I interferons are synthesized to induce the expression of antiviral molecules and interfere with virus replication. The importance of early antiviral type I IFN response against virus invasion has been emphasized during COVID-19 as well as in studies on the microbiome. Further, type I IFNs can directly act on various immune cells to enhance protective host immune responses to viral infections. However, accumulating data indicate that IFN responses can be harmful to the host by instigating inflammatory responses or inducing T cell suppression during virus infections. Also, inhibition of lymphocyte and dendritic cell development can be caused by type I IFN, which is independent of the traditional signal transducer and activator of transcription 1 signaling. Additionally, IFNs were shown to impair airway epithelial cell proliferation, which may affect late-stage lung tissue recovery from the infection. As such, type I IFN–virus interaction research is diverse, including host antiviral innate immune mechanisms in cells, viral strategies of IFN evasion, protective immunity, excessive inflammation, immune suppression, and regulation of tissue repair. In this report, these IFN activities are summarized with an emphasis placed on the functions of type I IFNs recently observed during acute or chronic virus infections.

## 1. Introduction

In 1957, Isaacs and Lindenmann reported that a molecule secreted from cells interfered with influenza virus replication. It was named “interferon” (IFN) to reflect its notable inhibitory activity against virus propagation [1]. There have since been three types of IFN defined. The type I IFN family is mainly composed of IFN-α subtypes (13 in humans and 14 in mice) and IFN-β; it also includes IFN-ɛ, IFN-τ, IFN-κ, IFN-ω, IFN-δ, and IFN-ζ of which functions are yet ill-defined [2,3,4]. Two other classes of IFNs, type II IFN (IFN-γ) and type III IFN (IFN-λ), have been identified and revealed to impair virus propagation. Almost every cell can synthesize and respond to type I IFNs. However, type II IFN (IFN-γ) is produced by specific immune cells such as T cells, natural killer (NK) cells, and innate lymphoid cells (ILCs) to regulate host innate and adaptive immune responses to virus infections [5,6]. Type III IFNs include IFN-λ1 (IL-29), IFN-λ2 (IL-28A), and IFN-λ3 (IL-28B), which have been characterized for their antiviral and immune regulatory activities, while the function of the recently identified IFN-λ4 is unclear [7,8]. Unlike type I IFNs, type III IFNs are known to predominantly act on mucosal epithelial cells and several subsets of immune cells due to the restricted expression of their cognate receptor. This review will focus on detailing the functionalities of type I IFNs.

The powerful antiviral function of type I IFNs has been proved in numerous experimental systems of tissue culture and animal models, as well as in patients suffering from virus infections [2,5]. Further, many pathogenic viruses appear to have tactics to evade or antagonize the production of type I IFN or components within the type I IFN receptor signaling pathway [9,10]. This emphasizes the importance of antiviral type I IFN responses in the host during virus infection. More recently, type I IFN was reported to be important for early host defense during severe acute respiratory syndrome coronavirus 2 (SARS-CoV-2) infection [11]. However, in contrast to the host-protective function of type I IFN, this cytokine can also contribute to immune dysregulation or tissue injury during virus infections [12]. These observations suggest that type I IFN operates in various ways depending on the timing, location, type of cells, and pathogens. As such, type I IFN can be both favorable and detrimental to the host.

## 2. Protective Functions of Type I IFN

### 2.1. Antiviral Activity

#### 2.1.1. Induction of Type I IFN Response

**Figure 1:**
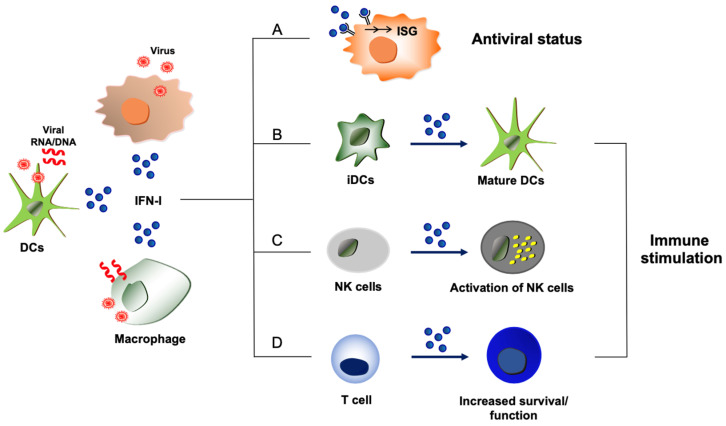
Host-protective and immune stimulatory functions of type I IFNs during virus infections. Type I IFNs (shown as IFN-I) are produced from virus-infected cells or immune cells such as dendritic cells (DCs) and macrophages that uptake viral components or dying cells that are infected by viruses. (A) Proteins derived from ISGs display antiviral activities and disrupt the replication processes of viruses, leading to the establishment of an antiviral state. (B) Exposure to type I IFNs induces the maturation of immature dendritic cells (iDCs) with upregulation of MHC molecules and co-stimulatory molecules, which can improve protective host immunity to virus infections. (C) Virus-induced type I IFNs can activate NK cells to enhance NK cell-mediated host immunity and elimination of viruses. (D) Type I IFNs can directly act on T cells to promote the survival or function of effector and memory T cells during virus infection.

Cells are equipped with innate sensors to detect foreign molecules, known as pattern recognition receptors (PRRs), that send an alarm to the host immune system [13]. This forefront innate immunity is manifested by potent antiviral IFN responses [13]. Multiple cellular PRRs have been identified, including retinoic acid-inducible gene-I (RIG-I)-like receptors (RLRs), toll-like receptors (TLRs), and DNA sensors such as cyclic GMP-AMP synthase-stimulator of interferon genes (cGAS-STING) alliance [14,15,16]. Once the PRRs detect the presence of pathogenic molecules, a specific signaling cascade is elicited to synthesize cytokines, such as type I IFN [14,15]. The binding of type I IFN to its cognate IFN receptor (IFNAR) triggers the well-known Janus kinase-signal transducer and activator of transcription (JAK-STAT) signaling pathway [17]. A complex composed of STAT1, STAT2, and interferon regulatory factor 9 (IRF9) (STAT1-STAT2-IRF9) is formed and acts on the IFN stimulatory response element (ISRE) to initiate the transcription of antiviral genes [17,18,19]. Proteins encoded by IFN-stimulated genes (ISGs) impair virus replication processes to establish an antiviral status [19,20] (Figure 1). Detailed signaling pathways for type I IFN production through diverse PRRs are not discussed in this review, as this review is focused on the functionality of type I IFNs.

#### 2.1.2. Viral Strategies to Subvert Type I IFN Response

Almost all viruses that are pathogenic in humans have been investigated for their interaction with type I IFN. Indeed, type I IFN has been shown to interfere with the replication of viruses in vitro and in animal models [21]. Development of an IFNAR1-deficient mouse model demonstrated that the loss of type I IFN signaling due to the lack of the responsive IFN receptor renders mice highly susceptible to virus infections [22]. Indeed, recombinant IFN-α2 and IFN-β have been clinically effective in treating patients infected with viruses, tumors, or immune diseases such as multiple sclerosis [23,24,25]. However, the mechanism for the therapeutic effects of type I IFN in patients infected with viruses is not clearly defined, as type I IFN could modulate the host defense and immunity in various ways, which is described further in other sections of this review.

Pathogenic viruses were shown to have multiple strategies to escape or counteract type I IFN responses, which enhances viral replication and dissemination [26,27,28]. The underlying mechanisms for viral evasion of type I IFN response have been extensively studied. For example, influenza A virus (IAV) evades the formidable type I IFN responses through various ways, which include nonstructural protein 1 (NS1) binding to double-stranded RNA (dsRNA); inhibition of RIG-I activation by NS1; mitochondrial antiviral-signaling protein (MAVS) inhibition by polymerase basic protein 1 (PB1), PB2, or PB1 frame 2 (PB1-F2); and IFNAR degradation by HA [29,30,31,32,33,34,35,36]. Many other viruses were also shown to impair the JAK STAT type I IFN signaling pathway, IFN receptor regulation, and/or functions of antiviral ISG products or molecules involved in the synthesis of type I IFN. Specific viral strategies to regulate type I IFN responses are innumerable and not further detailed in this review. The interference of type I IFN response creates a favorable environment for viral propagation. Current research continues to elucidate the viral mechanisms of immune evasion as well as host defense pathways against viral infection in order to better understand both viral pathogenesis and the host defense system.

#### 2.1.3. Type I IFN in COVID-19

Since the devastation of the severe acute respiratory coronavirus 2 (SARS-CoV-2) pandemic, type I IFN has garnered renewed interest as a research topic. Type I IFN responses have been investigated experimentally, as well as in the clinic in the context of coronavirus disease 19 (COVID-19). Notably, ineffective type I IFN responses were observed in critically ill COVID-19 patients when compared to cases of asymptomatic or mild SARS-CoV-2 infection [11,37]. This is partly attributed to the occurrence of genetic mutations in type I IFN pathways or the presence of autoantibodies against type I IFNs in those patients with severe COVID-19 [38]. The 2′5′-oligoadenylate synthetase (OAS) proteins are ISG products that act as sensors of dsRNA, which is often generated during virus replication processes. Thus, OAS plays a key role in host innate immunity against some viral infections [39]. Intriguingly, a splicing variant of the OAS1-encoding gene was found to be strongly associated with COVID-19 hospitalization risk in infected individuals [40]. The identification of these genetic risk factors for COVID-19 helps to emphasize the importance of type I IFN signaling in promoting host protection from viruses. However, type I IFN response was also correlated with increased inflammatory responses in severely ill COVID-19 patients in another study [41]. Clinical trials using recombinant type I IFN yielded various mixed results, with some studies showing little benefit from type I IFN therapy, while a few others reported that early or local administration of type I IFN was therapeutically effective [42,43]. It is likely that the type I IFN response is beneficial to the host only when and where the host antiviral mechanism is suppressed by massive viral amplification. It is also possible that the host protective antiviral activity of type I IFN is counterbalanced by type I IFN’s harmful effects, such as pathogenic inflammatory responses or other immune regulatory actions.

#### 2.1.4. Type I IFN from Microbiome

Another field of type I IFN study has extended to its association with the microbiome. Gut commensal microbiota were shown to regulate steady-state induction of type I IFN, increasing the host resistance to viral infections [44]. Depletion of the gut microbiota results in increased virus propagation and correlates with decreased expression of type I IFN in the lung upon infection with IAV or respiratory syncytial virus (RSV) [45]. Gut microbiota express ligands for PRRs, such as bacterial DNA and polysaccharide A that can trigger the type I IFN production pathway [46,47]. Further, metabolites produced from commensal bacteria, such as short-chain fatty acids (SCFAs) and secondary bile acids, were documented to induce the synthesis of type I IFNs and impair the spread of viruses [48,49]. These studies uncovered the role of microbiota in IFN responses and virus infections and provided a possible therapeutic option for controlling viral diseases through the utilization of endogenous IFN responses [50]. Further, the possible differences between the microbiota of animals housed in facilities at different locations could potentially affect the experimental outcomes of animal model studies [51,52]. Future studies remain to be conducted to increase the knowledge about the defined role of compositions of diverse microbiota regulating type I IFN response and the resultant effect on the host immunity to pathogenic infections.

### 2.2. Immune Stimulatory Action of Type I IFNs

#### 2.2.1. Type I IFN Production from Immune Cells

Type I IFN’s antiviral activity has been extensively studied in non-immune cells where many viruses propagate [17] (Figure 1). However, immune cells such as dendritic cells (DCs) can detect viral antigens not only in the infected cells but also by engulfing viral components or infected cells [53]. The innate immune cells express high levels of PRRs. Plasmacytoid DCs (pDCs) are specialized IFN-producing cells that synthesize the highest amount of IFNs (up to 10 pg) per cell [54,55]. The high basal level of IRF7 expressed in pDCs also helps pDCs to express type I IFN at levels 10–100 fold higher than other types of cells upon infection [56]. Since highly pathogenic viruses have various tactics of type I IFN evasion during virus replication in the infected cells, DCs such as pDCs often turn out to be the major cell type that produces type I IFN during infection in vivo.

#### 2.2.2. Immune Stimulation by Type I IFN

Type I IFNs are well known to stimulate or induce differentiation of several types of immune cells (Figure 1). For example, type I IFN is one of the major cytokines that activate natural killer (NK) cells, but it also increases the activation and maturation of fully committed DCs by increasing the level of MHC proteins of MHC-I and MHC-II and co-stimulatory molecules, including B7-1 (CD80), B7-2 (CD86), and CD40 [57,58,59]. The effect of type I IFN on T cells was initially considered controversial due to its anti-proliferative and apoptotic activity, which can be notably observed in vitro [60]. However, when the receptor for type I IFN was deleted exclusively on T cells, T cell survival and T cell memory responses were impaired during lymphocytic choriomeningitis virus (LCMV) infection in vivo [61,62]. This indicates that intact type I IFN signaling in T cells is crucial for optimized functions of effector and memory T cell responses. Interestingly, the results seemed to be dependent on the type of pathogen, as the expansion of T cells was not affected by the lack of the receptor for type I IFNs in T cells upon infection by vaccinia virus expressing LCMV glycoprotein [61]. This implies that other signaling cues could replace the IFN function or that a specific pathogen interaction with the host may determine the necessity of the IFN signaling for T cell function.

## 3. Pathogenic Functions of Type I IFN

### 3.1. Immune Suppression by Type I IFN

#### 3.1.1. Inhibition of Cellular Development Process by Type I IFN

**Figure 2:**
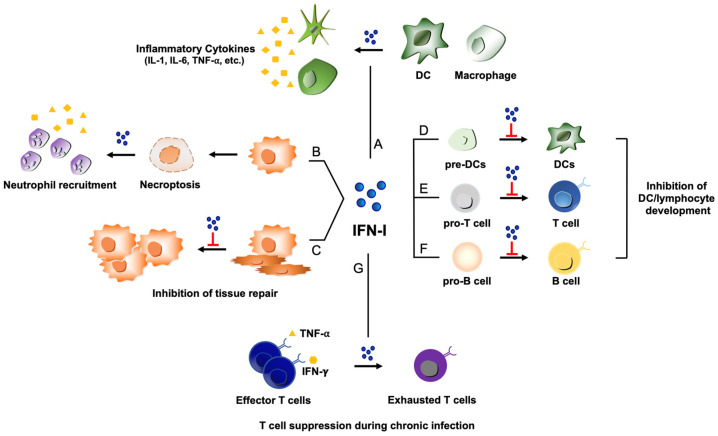
Potential pathogenic functions of type I IFNs during virus infections. (A) Type I IFN (shown as IFN-I) activates DCs and macrophages, which can lead to excessive production of pro-inflammatory cytokines such as IL-1, IL-6, and TNF-⍺ and contribute to detrimental inflammation. (B) ZBP-1 induced by type I IFNs can recognize viral nucleic acids to trigger necroptosis, resulting in the recruitment of neutrophils and subsequent inflammatory responses. (C) Type I IFN can impede proliferation of epithelial cells for tissue repair process during the stage of recovery from viral infections. (D, E, and F) Type I IFN displays a repressive activity on the developmental processes of several immune cells: Inhibition of DC development from DC precursors (pre-DCs) (D); suppression of T cell maturation from T cell progenitors (pro-T cells) (E); inhibition of B cell development from progenitor B cells (pro-B cells) (F). (G) Under the influence of type I IFN, virus-specific T cells can lose the effector function to transition into a state of exhaustion during chronic viral infections.

While type I IFN was shown to stimulate several types of terminally differentiated immune cells or further mature them, it appeared to inhibit the developmental processes of several immune cells, such as B cells, T cells, and DCs (Figure 2). Injection of mice with recombinant type I IFN blocked B cell development at the stage of pro-B cells, which was due to apoptosis of B cell progenitors [63,64]. Interestingly, type I IFN-induced apoptosis of B cell progenitors was independent of STAT1. Similarly, IL-7-mediated survival of progenitor T cells was impaired by type I IFN via a STAT1-independent mechanism [65,66]. Further, type I IFN was shown to inhibit DC development processes in the granulocyte-macrophage colony-stimulating factor (GM-CSF) or Fms-like tyrosine kinase 3 ligand (Flt3-L)-supplemented bone-marrow-derived DC culture system [67,68]. Importantly, measles virus or LCMV infection-induced type I IFN was shown to inhibit the development of CD11c+ DCs from bone marrow cells. Flt3-L-mediated amplification and expansion of CD11c+ DCs in mice was substantially impaired during infection by an immune suppressive strain of LCMV. The inhibition of DCs by type I IFNs was still observed in the absence of STAT1, STAT4, or STAT6, whereas type I IFNs could not block DC development when bone marrow cells were lacking STAT2. Thus, the DC inhibitory pathway utilized a STAT1-independent but STAT2-dependent unique IFN signaling pathway [67]. It is conceivable that the host has a mechanism to use this non-traditional IFN signaling pathway, which is independent of STAT1, to maintain the homeostasis of several immune cells. The pathway may be exploited by some immune regulatory pathogenic viruses to escape the local immune response and promote long-term viral persistence.

#### 3.1.2. Suppression of Host Immunity during Virus Persistence

The LCMV variant Clone 13 (Cl 13) infection model has been widely used for revealing mechanisms for T cell suppression and virus persistence [69,70,71]. Following LCMV Cl 13 infection, virus-specific T cells develop but gradually lose their functionalities, as evidenced by the substantially diminished capabilities to produce antiviral cytokines, such as IFN-γ and TNF-α, to display cytotoxic activity with granzymes and perforin secretion, as well as to proliferate in response to antigenic restimulation. These exhausted T cells fail to eradicate viruses, which allows viruses to establish persistence in the host. Molecules that are crucial for the T cell exhaustion during virus persistence have been identified in this model, including programmed cell death protein 1 (PD-1), T cell immunoglobulin and mucin domain-containing protein 3 (Tim-3), and lymphocyte activation gene 3 (LAG-3) [72,73,74,75,76]. Intriguingly, blockade of type I IFN signaling using anti-IFNAR1 antibody during LCMV Cl 13 chronic infection relieved T cell suppression and led to accelerated clearance of viral infection [77,78]. This suggests that type I IFN response contributes to T cell inhibition and virus persistence during an immune suppressive LCMV Cl 13 infection (Figure 2). Type I IFNs can also increase the expression of programmed cell death ligand 1 (PD-L1) and immune regulatory cytokine of IL-10 that can affect immune suppression during virus infections. Eradication of LCMV Cl 13 was dependent on CD4+ T cell responses, as CD4+ T cell depletion abrogated the protection that was conferred by the IFN blockade. However, IFN signaling on T cells is not critical for virus elimination. Removal of IFNAR1 from CD11c+ or CD4+/CD8+ T cells caused an increase in the titer of viruses, which persisted throughout the infection [79]. The absence of IFNAR1 expression on B cells did not lead to any notable improvements in the clearance of Cl 13 infection. On the other hand, type I IFN signaling on NK cells was shown to be crucial for killing CD4+ and CD8+ T cells during LCMV Cl 13 infection [79]. In the absence of IFNAR1 on NK cells, the follicular helper T cells, germinal center reaction, LCMV-specific B cell responses, and functional LCMV-specific cytotoxic T lymphocytes (CTLs) were improved, thus eliminating LCMV in mice [79]. Therefore, the immune stimulatory function of type I IFN on NK cells appeared to dampen the host immunity to fight against chronic LCMV Cl 13 infection. This series of studies illustrates the significance of interactions among immune cells and the role of type I IFN in immune regulation in the context of persistent pathogenic infections.

The immune suppressive function of type I IFN was extended to human immunodeficiency virus (HIV) infection in animals, which was conducted in multiple studies. Zhen et al. showed that blockade of IFNAR in combination with antiretroviral therapy accelerated viral clearance and immune activation in a humanized mouse model of chronic HIV-1 infection in vivo, as the treatments reduced the viral reservoir, decreased T cell exhaustion, and restored HIV-1-specific CD8+ T cell functions [80]. Moreover, in another study using the humanized mouse model, blocking IFNAR resulted in a decrease in HIV-1-induced apoptosis of CD4+ T cells and enhanced function of various types of human T cells, including HIV-1-specific CD8+ and CD4+ T cells [81].

### 3.2. Inflammation and Tissue Injury Caused by Type I IFN

#### 3.2.1. Inflammation

While the immune stimulatory function of type I IFN could contribute to protective immunity during infection or vaccination, it may exacerbate harmful inflammatory responses and cause collateral tissue damage during virus infection or even after viral clearance. Therefore, inducing and maintaining balanced host immunity is critical for the outcome of protective immunity. A good example is the use of monophosphoryl lipid A (MPL), a detoxified lipopolysaccharide (LPS), as an adjuvant. MPL was developed to alleviate the pathogenic inflammatory function of the representative TLR4 ligand, LPS, and is currently used in humans for vaccination against infectious diseases [82].

Type I IFN can increase the production of pro-inflammatory cytokines and chemokines from monocytes and macrophages as well as other lymphocytes (Figure 2). Low levels of pro-inflammatory cytokines were observed when mice that are defective in type I IFN receptors were exposed to TLR agonists, demonstrating the causal link between type I IFN and pro-inflammatory cytokines [83]. Although the initial increase in the cytokines and chemokines could send the danger signal of pathogenic infections to the host immune system and protect against the invading microbe, uncontrolled and sustained production of cytokines could damage the tissue over time, which can become detrimental to the host. Excessive secretion of cytokines, which is called a cytokine storm, has often been correlated with robust replication of highly pathogenic influenza viruses. This suggests that viral propagation produces more viral components that interact with an increased number of immune cells and continue to stimulate immune cells for the ensuing production of cytokines. Serious inflammation was observed when animals were infected by re-constructed 1918 pandemic influenza viruses compared to seasonal influenza virus infection [84,85,86]. Similar observations were made when patients were infected by highly pathogenic influenza virus strains such as H5N1. The possible roles of influenza viral proteins in the cytokine storm have been reported. Avian adaptive mutations in the viral PB2 polymerase gene are found in highly pathogenic avian H5N1 and 1918 pandemic influenza viruses and are responsible for the increase in the level of aberrant viral RNAs that can over-stimulate host innate immunity to result in pathogenic inflammation [87]. Further, 1918 influenza viral NS1 was shown to induce exaggerated cytokine responses, although it blocks ISG transcription [88]. Additionally, 1918 viral NS1 interacts with host protein CRK as well as the p85β subunit of phosphoinositide 3-kinase (PI3K) to activate cellular signaling and increase virulence, which may contribute to the dysregulation of host innate immunity [89]. Inflammatory responses can remain even after infectious viruses are not detectable. It is possible that the harmful inflammatory responses are sustained due to the residual viral components and viral RNAs within the host after pathogenic viruses are cleared. Further, the cytokines and chemokines recruit immune cells to the target tissue where their antimicrobial toxic products and cytokines exert actions that may last for some time even after infectious viruses are eliminated [90].

The pro-inflammatory effects of type I IFN have been studied in mouse models of coronaviruses. In BALB/c mice infected with SARS-CoV-1, there was a delayed but significant type I IFN response that was responsible for the accumulation of inflammatory monocytes and macrophages in the lungs. The process caused fatal pneumonia with leaking blood vessels, while mice deficient in the receptor for type I IFNs survived the infection, supporting the crucial role of type I IFN in the inflammatory pathogenesis during virus infections [91]. However, in parallel experiments using influenza and mouse hepatitis virus (MHV-1), IFNAR-deficient mice were more susceptible and succumbed to sub-lethal doses of these viruses, suggesting that IFN signaling is protective and possibly triggers an antiviral pathway, contrary to the observations made during SARS-CoV-1 infection [91]. In a recent study using IFNAR-deficient or (IRF3 and IRF7) double knockout mice during SARS-CoV-2 infection, type I IFN signaling was shown to be crucial for the recruitment of inflammatory cells to the lungs [92]. Up-regulation of many antiviral ISGs and monocyte recruiting chemokines were not observed in the IFNAR-deficient mice or (IRF3 and IRF7)-deficient mice during infection by SARS-CoV-2 [92], indicative of the inflammatory function of type I IFN during virus infections. Studies have shown that SARS-CoV-2 viral proteins can activate inflammatory reactions. For example, viral protein encoded by ORF3a induces IL-1β secretion and inflammasome activation [93]. Viral spike (S) protein triggers inflammatory responses via a TLR2-dependent NF-κB signaling pathway [94]. Further studies remain to be conducted to define the underlying mechanisms and possible involvement of type I IFN in the pathogenic inflammation during SARS-CoV-2 infection.

Z-DNA-binding protein 1 (ZBP1) is a type I IFN-inducible protein that mediates inflammatory cell death, such as necroptosis, upon influenza virus infection [95] (Figure 2). Recognition of influenza viral Z-RNAs by ZBP1 can induce activation of the receptor-interacting serine/threonine protein kinase 3 (RIPK3), which leads to stimulation of the necroptosis pathway mediated by mixed lineage kinase domain-like protein (MLKL). MLKL deletion from mice partially protected mice from influenza-induced mortality. While the virus titer and CD8+ T cell immunity did not change, neutrophil responses were attenuated in the absence of MLKL [96]. This suggests that ZBP1 recognition of viruses could induce MLKL necroptosis, which leads to the recruitment of neutrophils to the lungs and can be pathogenic to the host due to increased inflammation. However, MLKL-induced necroptosis could also display antiviral activity. More recently, a new pathway of inflammatory cell death, known as PANoptosis, as it is not explained by pyroptosis, apoptosis, or necroptosis alone, has been shown to be mediated by ZBP1 in the lungs of mice infected with coronaviruses [97]. It warrants further exploration for defining the role of these cell death mechanisms during virus infections and for determining how type I IFNs regulate these cell death pathways and associated inflammatory reactions or antiviral activities upon pathogenic virus infections.

Obviously, the inflammatory response is not solely dependent on type I IFN. It is unclear to what extent type I IFN is responsible for the pathogenic inflammatory responses during virus infections. Evaluation is made more difficult due to IFN’s ability to inhibit virus replication, which in turn may decrease the amount of viral nucleic acids that can stimulate immune cells. Thus, the effect of the type I IFN-mediated inflammatory response on the host defense and viral diseases could be strongly affected by the immune regulatory environment uniquely set up during specific virus infections.

#### 3.2.2. Impairment of Tissue Repair by Type I IFN

Type I IFNs were shown to inhibit epithelial cell proliferation during lung repair following influenza virus infection [98] (Figure 2). Adoptive transfer of wild-type bone marrow cells into IFNAR1-deficient mice demonstrated that receptor deficiency in the stromal compartment was sufficient to increase the proliferation of EpCam + MHC-II + CD49f^low^ type II alveolar epithelial cells at the late stage of virus infection [98]. Therefore, IFN could not only increase inflammation but also impair the regeneration of lung airway epithelial cells during the late stage of virus infections. Interestingly, at the late stage of infection, type I IFN was not detectable, suggesting that the local residual amount of type I IFN could be enough to cause the inhibition of epithelial cell proliferation. Since many antiviral proteins induced by IFNs are designed to block cellular proliferation to inhibit virus replication, their functions are likely to interfere with tissue repair during the recovery stage [18,21].

## 4. Perspectives

With advanced scientific knowledge and modernized techniques, the multifaceted functions of type I IFN have been uncovered. Although recombinant IFN-α2 and IFN-β were empirically used for the treatment of diseases in humans, such as chronic hepatitis C virus infection and multiple sclerosis, these therapeutics displayed limited efficacies, presumably due to the lack of specificity and the numerous functions of type I IFN. Thus, from the standpoint of therapeutic development, it would be prudent to determine how to tilt the balance towards the beneficial action of type I IFN and nullify or minimize the untoward activity of type I IFN during virus infections. This may be achieved by targeting a specific molecule downstream of the type I IFN pathway triggered through interaction between viruses and host defense. Therefore, continuous efforts to reveal the positive and negative functions of type I IFN during diverse virus infections will be necessary prior to the development of more efficacious therapeutics.
